# Healthcare Workers’ Attitudes Toward Older Adults’ Nutrition: A Descriptive Cross-Sectional Study in Italian Nursing Homes

**DOI:** 10.3390/geriatrics10010013

**Published:** 2025-01-16

**Authors:** Milko Zanini, Gianluca Catania, Marco Di Nitto, Lara Delbene, Stefania Ripamonti, Maria Emma Musio, Annamaria Bagnasco

**Affiliations:** 1 Department of Health Sciences, University of Genova, 16132 Genoa, Italy; gianluca.catania@unige.it (G.C.); marco.dinitto@unige.it (M.D.N.); lara.delbene@edu.unige.it (L.D.); annamaria.bagnasco@unige.it (A.B.); 2 BC Diet, ASST della Brianza Servizio Dietetico P.O. di Desio U.O.S.D. Malattie Endocrine, del Ricambio e della Nutrizione, 20832 Palermo, Italy; stefania.ripamonti@asst-brianza.it; 3 ASL 3 Genovese, 16125 Genova, Italy; mariaemma.musio@edu.unige.it

**Keywords:** nutrition of older people, malnutrition intervention, healthcare workers’ attitudes, nursing home care

## Abstract

**Background:** Malnutrition is a widespread issue among older people, significantly impacting health outcomes. Nutritional interventions can improve health, but their success often depends on the attitudes and knowledge of healthcare workers. **Aim:** This study assesses healthcare workers’ attitudes toward older people’s nutrition using the validated Italian version of the Staff Attitudes to Nutritional Nursing Geriatric care scale (SANN-G), focusing on staff in nursing homes in Northern Italy. **Methods:** A cross-sectional study was conducted with 1789 healthcare workers from 41 facilities. The SANN-G questionnaire measured attitudes across five dimensions: nutritional norms, habits, assessment, intervention, and individualization. Data were collected online and on paper, with descriptive and inferential statistical analyses (chi-square and ANOVA) performed using R software (R-4.4.2 for Windows). **Results:** Most healthcare workers were female (68.59%) and aged 41–50 (33.31%), with nursing assistants comprising 35.83%. Only 23.48% scored positively on the SANN-G scale, with most exhibiting neutral or negative attitudes. Healthcare workers who attended a malnutrition course were more likely to exhibit positive attitudes toward nutrition, particularly in the dimension of norms, assessment, intervention, and individualization. Younger healthcare workers, with the role of nurses and who attended a course on malnutrition, were more likely to have positive attitudes, while older healthcare workers and with the role of physicians tended to show neutral or negative attitudes. **Conclusions:** Education on malnutrition improves healthcare workers’ attitudes toward older adults’ nutrition, especially among younger nurses. The low percentage of positive attitudes (23.48%) underscores the need for broader education programs to enhance nutritional care quality.

## 1. Introduction

Malnutrition is a widespread issue among older people across various care settings, significantly impacting health outcomes. Studies have shown that the prevalence of malnutrition varies depending on diagnostic criteria and care settings. For instance, research indicates that up to 18.2% of nursing home residents are malnourished, with higher rates observed when age-specific criteria are used. Prevalence ranges from 3.8% to 18.2%, depending on the diagnostic criteria, such as BMI < 20 kg/m^2^ or combinations with weight loss and reduced intake. Using age-specific BMI (e.g., <22 kg/m^2^ for those ≥ 70 years) can double these rates, reflecting age-related declines in muscle mass [1]. Additionally, among hospitalized older people, the risk of malnutrition is strongly associated with higher mortality rates and longer hospital stays [2].

Nutritional interventions effectively address protein-energy malnutrition in older adults, improving health outcomes. Dietary counseling combined with oral nutritional supplementation (ONS) is the most effective approach to increasing energy intake and body weight [3]. Specifically, previous systematic reviews highlight the benefits of protein-energy supplementation, which enhances energy and protein intake in most cases [4,5]. Additionally, combining supplements with environmental and organizational interventions improves nutritional, anthropometric, and functional outcomes. An analysis of nine RCTs demonstrated that targeted nutritional strategies boost muscle strength and reduce mortality in malnourished older adults or those at risk [6]. These findings emphasize the importance of personalized nutritional interventions tailored to individual needs, addressing both physical and contextual factors. Furthermore, short-term protein supplementation during rehabilitation improved dietary protein intake and physical recovery, although more robust studies are needed to confirm these findings [7].

The role of caregivers and healthcare workers is crucial to the success of nutritional interventions. Education for healthcare workers can significantly enhance nutritional care practices. For example, an interprofessional approach that includes staff education has been shown to improve nutritional outcomes in hospitals and long-term care facilities [8]. Additionally, patient nutritional education combined with physical exercise has been found to be more effective than nutritional interventions alone in improving frailty and physical performance in older adults [9].

Several studies have highlighted gaps in nutritional care due to inadequate education among healthcare workers [10,11,12]. Poor nutritional knowledge and negative attitudes toward nutritional care are common issues. A study on the nutritional status of older people in geriatric rehabilitation revealed that malnutrition is often inadequately addressed, partly due to insufficient education among healthcare workers [13]. Moreover, a systematic review found that nutritional interventions in hospitals and long-term care facilities are more effective when combined with comprehensive staff education and an interprofessional approach [8].

The role of continuing education on malnutrition is emphasized in recent work by Castaldo and Patel [14,15], which identified a strong link between malnutrition interventions and healthcare worker education. These studies found that healthcare workers with specialized education in malnutrition had significantly more positive attitudes toward personalized care, reinforcing the idea that education is essential for effective interventions. However, the study also highlighted persistent gaps in nutritional knowledge and the need for broader educational initiatives to ensure consistency in care across different healthcare settings.

Furthermore, Johnston [12] demonstrated that structured educational programs for healthcare workers in dementia wards significantly improved the nutritional status of residents in care facilities. Their findings align with our observations during the study, emphasizing the need for practical learning approaches that incorporate tools like the Mini Nutritional Assessment (MNA) [16] and food diaries. These tools not only enhance care but also help healthcare workers better understand and address the unique nutritional challenges faced by older people.

Despite the focus on interventions such as ONS supplementation and integration, there remains a gap in addressing the attitudinal competencies of healthcare workers regarding malnutrition. To be effective, interventions should integrate both knowledge-based and attitudinal education to ensure that healthcare workers can confidently address malnutrition in frail older people [17].

Therefore, this study aims to evaluate healthcare workers’ attitudes and practices toward older peoples’ nutrition in nursing homes using the Italian version of the Staff Attitudes to Nutritional Nursing Geriatric Care Scale (SANN-G).

## 2. Materials and Methods

### 2.1. Study Design

This was a cross-sectional study with the application of the Italian version of the Staff Attitudes to Nutritional Nursing Geriatric care scale (SANN-G), developed by Bonetti [18], which has been validated for use in Italian healthcare settings. The STROBE checklist for observational studies was followed for the reporting of this study [19].

### 2.2. Setting and Participants

The study focuses on healthcare workers employed in nursing homes in Northern Italy. A convenient and consecutive sample of healthcare workers from 41 nursing homes in Northern Italy was invited to participate. Healthcare workers from various professional roles, including nurses, nursing assistants, and physicians, were recruited. Participation was voluntary, and respondents were assured of the anonymity of their responses. The inclusion criteria for healthcare workers were:

(1) employment in a healthcare role within a nursing home and
(2) direct provision of care to older residents.

No exclusion criteria were applied based on professional roles, including all care workers employed in nursing homes.

### 2.3. Data Collection

Data were collected between 2019 and 2023. All participants provided written informed consent before completing the questionnaire. The survey was designed to take approximately 20 min, and healthcare workers were given adequate time to provide accurate and thoughtful responses.

The survey was administered both online and in paper format, depending on healthcare workers’ preference. The online survey was distributed via a secure link to the “Sondaggi.unige.it” server (access from 1 June 2019 until 30 May 2023), which ensured encrypted and protected data acquisition. Paper versions were made available at the workplace and collected by designated coordinators. Both methods ensured healthcare workers’ anonymity, with no identifying information being requested or recorded. All survey responses, both online and paper-based, were digitized and compiled into a single dataset for analysis.

### 2.4. Variables

The sociodemographic characteristics of the healthcare workers were collected, including nursing home affiliation, sex, age, and years of experience. Additionally, healthcare workers were asked about their role in the nursing home (e.g., physician, nurse, nursing assistant, or other) and whether they had attended a specific educational course on malnutrition. For the latter, this refers to a recognized course on the proper management of malnutrition for older people, such as those offered by universities, regional authorities, or accredited providers. The course on malnutrition could also fall within the scope of Continuing Medical Education. In Italy, all healthcare workers are required to take these courses (participants can choose which courses to attend) to demonstrate that they have the most up-to-date knowledge in their field and according to the setting in which they work.

Healthcare workers’ attitudes toward nutritional care were measured through the SANN-G questionnaire, which evaluates healthcare workers’ attitudes, knowledge, and practices regarding older people’s nutrition. This questionnaire consists of 18 items with a five-option Likert-type Scale (1 = completely agree, 2 = agree, 3 = doubtful, 4 = disagree and 5 = completely disagree). The cut-off score for negative attitudes is <54 points, while a score ≥ 72 reflected positive attitudes. A cut-off score for positive attitudes can also be obtained for each dimension. The questionnaire includes several sections covering key areas such as (cut-off for positive attitudes in brackets):

- Norms: Attitudes toward nutritional standards and guidelines in older people’s care (≥20);
- Habits: Routine nutritional practices and interventions (≥16);
- Assessment: Approaches to nutritional screening and monitoring (≥16);
- Intervention: Strategies for managing malnutrition and associated nutritional risks (≥12);
- Intervention: Interventions needed to manage the disorders linked to malnutrition (≥12);
- Individualization: Adaptation of nutritional care to meet the individual needs of patients (≥8).

### 2.5. Sampling

The required sample size for this cross-sectional study was calculated using the formula for estimating proportions [20]. The calculation considered a 95% confidence interval (Z = 1.96), an estimated proportion of nurses with positive attitudes toward nutritional care of 39.2% based on previous research in a large cohort [21], and a precision level of 5% (d = 0.05). Using these parameters, the minimum required sample size was determined to be 366 healthcare workers.

### 2.6. Data Analysis

Data analysis was performed using the statistical software R (R-4,4,2 for Windows) [22], an open-source environment widely used for statistical computation and data analysis. Missing data in SANN-G responses were handled with listwise mean imputation. Descriptive statistics were calculated to summarize the demographic characteristics of the sample, such as gender, age, professional role, and years of experience in older people’s care.

In addition to descriptive statistics, inferential analyses were conducted to examine the relationships between healthcare workers’ demographic characteristics and their attitudes toward older people’s nutrition. Chi-square tests were used to assess associations between categorical variables and the SANN-G scores. T-tests and one-way analysis of variance (ANOVA) were employed to compare the means of SANN-G global scores across all categorical variables considered. For ANOVA, the Bonferroni post hoc test was performed to explore differences between categories. A Levene’s test was used to assess whether the assumption of equality of variances was met before conducting these analyses. In cases of unequal variances, the Welch t-test and Welch ANOVA were applied.

A hierarchical logistic regression analysis was conducted to examine the factors associated with healthcare workers’ global attitude toward older people’s nutrition. The SANN-G global score was dichotomized to identify healthcare workers who reported “negative or neutral attitudes” or “positive attitudes” toward older people’s nutrition (negative or neutral attitudes = 0, positive attitudes = 1). Multicollinearity (measured through the Variance Inflation Factor—VIF) and the Hosmer–Lemeshow test were checked before conducting the analysis. The significance level was set at *p* < 0.05.

Scatter plots were generated to visually assess the distribution of responses across the main attitudinal dimensions, including norms, habits, assessment, intervention, and individualization. Additionally, contingency tables were used to explore interactions between variables such as professional education on malnutrition and corresponding attitudinal scores. The results of these analyses were used to identify trends and outliers, providing a comprehensive understanding of healthcare workers’ attitudes toward older people’s nutritional care.

### 2.7. Ethical Approval

This study was conducted in accordance with the ethical standards outlined in the Declaration of Helsinki. Approval for the study was obtained from the Regional Ethics Committee of Liguria (ID 11116—No. 677/2020; approval date: 21 December 2020). All healthcare workers provided informed consent prior to participation, and no personally identifiable information was collected.

## 3. Results

### 3.1. Demographic and Professional Characteristics

The analysis of the SANN-G project, conducted across 41 nursing homes with a sample of 1789 healthcare workers (98% response rate), primarily aimed to examine healthcare workers’ attitudes toward older people’s nutrition. Descriptive statistics revealed that the majority of healthcare workers were female (68.59%) and aged between 41 and 50 years (33.31%). Various professional roles were represented, with nursing assistants comprising the largest group (35.83%) (Table 1).

### 3.2. Attitudes Toward Older People’s Nutrition by SANN-G Dimensions

We chose to use scatter plots to visually illustrate the distribution of attitudes, allowing for easy identification of patterns, outliers, and general trends. Scatter plots help in understanding the concentration of attitudes across different scales, revealing nuances in responses that simple statistical tables might not capture.

In the SANN-G analysis, scatter plots were employed to visually represent the distribution of attitudes across various study dimensions, including nutritional norms, habits, assessment, intervention, and individualization. These plots provided a clear visual differentiation among healthcare workers with positive, negative, or neutral attitudes, highlighting where the majority of the sample fell within each category.

**Figure 1:**
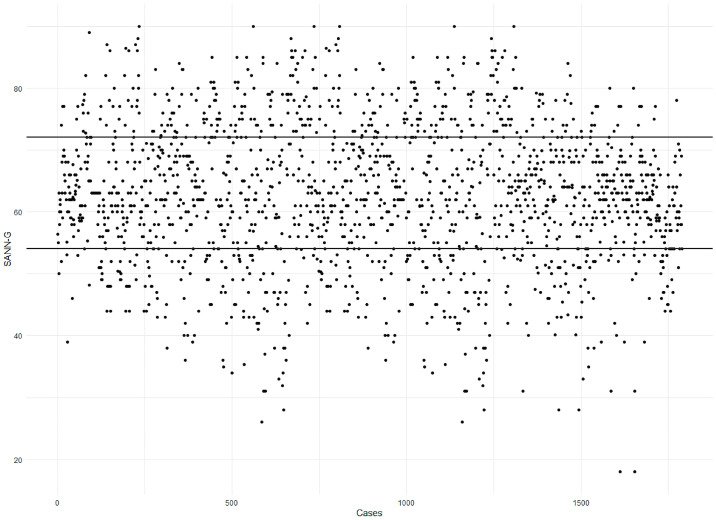
Overall distribution of responders’ global score in SANN-G; over the 72 scoreline, they reached a positive attitude, under the 54 scoreline they reported negative attitudes, and between the two scores they reported neutral attitudes.

Attitudes were measured across several dimensions, such as norms, habits, assessment, intervention, and individualization. Positive attitudes toward personalization and individualized care for older people were particularly prevalent among younger respondents and healthcare workers who had received specialized education. However, only 23.48% of healthcare workers achieved an overall positive score on the SANN-G scale, indicating that a significant portion of the sample still held neutral or negative attitudes toward older people’s nutritional care (Figure 1).

The scatter plot for nutritional standards revealed that only a small percentage of healthcare workers (13.36%) scored positively (score ≥ 20), with the majority reflecting more neutral or negative attitudes. In contrast, for habits, half of the healthcare workers (50.42%) demonstrated positive attitudes (score ≥ 16), suggesting a more balanced view in this area. In the analysis of assessment, fewer than half (40.02%) of the healthcare workers achieved a positive score (score ≥ 16), indicating that the majority held more conservative or neutral views regarding nutritional assessment practices.

In terms of intervention, the scatter plot showed that approximately half of the healthcare workers (48.69%) achieved a positive score (score ≥ 12), indicating mixed opinions on the application of active intervention strategies. However, the individualization dimension stood out, with more than half (54.95%) of healthcare workers scoring positively (score ≥ 8), suggesting stronger support for personalized care in older people’s nutrition.

### 3.3. Associations Between Sociodemographic Characteristics and Attitudes

Contingency tables in the SANN-G analysis were used to examine the relationships between healthcare worker characteristics (such as gender, age, professional role, and whether they had completed specific malnutrition education) and their attitudes toward older people’s nutritional care. These tables provided insights into the statistical significance of these relationships and highlighted key demographic and professional factors influencing attitudes.

The contingency table (Table 2) indicated that there is no significant difference in responses based on the gender of healthcare workers. However, a significant difference emerges with respect to age. Healthcare workers aged 31–40 and 51–60 years exhibited a significantly higher percentage of positive attitudes towards nutrition guidelines (*p* = 0.002). Additionally, a variation in responses was observed based on professional roles. Nurses and other healthcare professionals, compared to physicians and support staff, exhibited a significantly higher percentage of positive attitudes toward the guidelines (*p* < 0.001). Furthermore, completing a course on malnutrition had a significant impact, as those who participated in such a course reported a significantly higher percentage of positive attitudes toward the guidelines (*p* < 0.001). Contingency tables for each single dimension of the SANN-G scale and mean difference of SANN-G global score are reported in Supplementary Materials.

### 3.4. Factors Associated with Positive Attitudes: Regression Analysis

The results of the hierarchical logistic regression analysis (Table 3) indicated that some factors are associated with positive attitudes toward nutritional care. Nurses (OR: 2.42; 95% CI: 1.66–3.55; *p* < 0.001) and other healthcare workers, such as physicians or logopedics (OR: 2.14; 95% CI: 1.37–3.31; *p* < 0.001) were more likely than physicians to have positive attitudes toward older adults’ nutrition. Moreover, healthcare workers who followed a training course on malnutrition were more likely to have positive attitudes toward older adults’ nutrition (OR: 1.54; 95% CI: 1.16–2.03; *p* < 0.002). No differences were found regarding the sex and age of healthcare workers.

## 4. Discussion

The aim of this study was to assess healthcare workers’ attitude toward older people’s nutrition in nursing homes. This study analyzed healthcare workers’ attitudes toward older adults’ nutrition in nursing homes (long-term care facilities), examining the role of courses and programs focused on nutrition and malnutrition. The findings align with a growing body of international research that underscores the critical role of healthcare worker training in addressing malnutrition among older people—a persistent global public health challenge [3,7]. The study by Castaldo et al. [14] further supports the importance of continuing education, showing that healthcare workers who had received specific malnutrition education exhibited significantly more positive attitudes toward nutritional interventions compared to those without such education. Similar to our findings, the majority of healthcare workers initially displayed neutral or negative attitudes toward older people’s nutrition, but those with a formal education demonstrated a clear shift toward more proactive and personalized care. This is consistent with the evidence from Johnston [12] which highlighted the role of structured education in improving nutritional outcomes for patients with dementia. Their study showed that professionals learned to better assess and address nutritional deficiencies using tools like the MNA and detailed food diaries, leading to improved patient energy intake and nutritional status.

Our analysis revealed significant disparities in attitudes toward older people’s nutrition based on factors such as professional role, age, and prior training, although age resulted in a non-significant association with positive attitudes toward nutritional care in a multivariable analysis. We discovered that sensitive interventions can lead to better quality of life for patients and cost savings in nursing homes [23,24]. Notably, healthcare workers who had undergone specific training in malnutrition exhibited significantly more positive attitudes toward nutritional care, particularly in areas like assessment, intervention, and individualization. Moreover, multivariable analysis showed that healthcare workers who attended a training course on malnutrition were 54% more likely to have positive attitudes toward older people’s nutrition. These results are consistent with prior studies [12,14] that highlight the transformative potential of education in enhancing healthcare practices. Younger healthcare workers and nursing staff were found to have more favorable attitudes toward proactive nutritional intervention in univariable analysis, emphasizing the importance of integrating education on malnutrition early in a professional’s career.

Moreover, our findings suggest that professional roles and demographic factors, such as age and experience, could play a significant role in shaping attitudes toward older people’s nutrition. For example, physicians were found to have less favorable attitudes toward personalized nutritional interventions compared to nurses and healthcare assistants, indicating the need for more interdisciplinary collaboration and education. Specifically, nurses and other healthcare workers were twice as likely to have positive attitudes toward malnutrition compared to physicians, according to multivariable analysis. The study conducted by Thomson [25] adds a critical dimension to this discussion. Their systematic review examined the effectiveness of ONS for frail older people, finding that outcomes often depended on staff education levels and the consistency in applying intervention protocols. While nutritional supplements showed potential benefits in terms of energy intake and mobility, their research implies that a successful intervention hinged on the healthcare workers’ ability to effectively implement the interventions, something that can only be achieved through comprehensive and ongoing education. Without adequate education, even well-designed nutritional programs may fail to yield significant results.

Multidisciplinary approaches, as demonstrated in the study by Beck [26], further highlight the need for education that involves various healthcare roles. Their randomized trial showed that the involvement of dietitians, physiotherapists, and occupational therapists in older people’s care led to significant improvements in quality of life, muscle strength, and oral care. The success of these interventions underscores the importance of interdisciplinary education, ensuring that all healthcare team members are aligned in their approach to nutritional care. This mirrors our findings, where trained staff were more engaged and displayed a greater inclination toward individualized and collaborative care strategies.

However, it is important to acknowledge the limitations within the existing body of evidence. For example, Brown and Copeman [27] did not find a statistically significant relationship between increased nutritional knowledge and actual improvements in residents’ diets. This suggests that while education is critical, it must be accompanied by strategies that translate knowledge into sustained practices. In some cases, repeated exposure to education and practical tools, such as food diaries or nutritional assessments, may be necessary to reinforce behavioral changes among healthcare workers [12].

In countries facing rapid population aging, healthcare systems must prioritize education on malnutrition for healthcare workers in geriatrics. Policymakers should consider allocating resources to education programs that specifically address nutritional care, an area that is often overlooked in favor of other medical interventions. Ongoing research, such as the Family conferences and shared prioritisation to improve patient safety in the frail elderly (COFRAIL) trial [28], underscores the potential for involving families and caregivers in nutritional interventions. This trial aims to evaluate whether shared decision-making through family conferences can improve patient safety and care outcomes for frail older people. Although still in progress, the study reflects a growing awareness that education should not be limited to healthcare workers but should also extend to families and caregivers to ensure a more holistic approach to care. Integrating families into the care process could amplify the effects of professional education, fostering a shared commitment to improving nutritional health outcomes.

This study highlighted the role of education on malnutrition in better attitudes and practices of healthcare workers in the nutritional care of older adults. Implementing specialized training programs can foster a more proactive and effective approach, promoting personalized interventions and significantly enhancing the quality of care. Moreover, individualizing nutritional care, supported by tools such as nutritional assessments and food diaries, can better address the complex needs of frail older adults. Interdisciplinary collaboration among different professional roles, such as nurses, assistants, and dietitians, is essential to ensure a cohesive and effective approach. Future studies should assess the impact of healthcare workers’ attitudes toward older adults’ nutrition in improving patient outcomes, specifically quality of life and malnutrition prevention.

## 5. Conclusions

This study demonstrated that education on malnutrition significantly influences healthcare workers’ attitudes toward older adults’ nutrition, particularly in areas such as nutritional norms, assessment, intervention, and individualization. The findings indicate that younger healthcare workers and those with a nursing role tend to have more positive attitudes compared to older respondents, physicians in particular. However, the overall low percentage of positive attitudes (23.48%) highlights the need for broader implementation of education and training programs across all professional roles. These results suggest that integrating continuing education on malnutrition into professional development frameworks is essential for enhancing the quality of nutritional care in nursing homes.

## 6. Limitations

Several limitations should be considered. Firstly, the cross-sectional design restricts the ability to establish causal relationships between healthcare workers’ educational backgrounds and their attitudes toward older adults’ nutrition. Additionally, the study sample was characterized by a convenience sample, which could limit the generalizability. However, data were collected from a large cohort including 41 healthcare facilities in 6 out of 21 Italian regions. Another limitation is the potential for social desirability bias, as healthcare workers might have provided responses they deemed socially acceptable rather than reflecting their genuine attitudes and practices. This bias was controlled by collecting anonymous answers, allowing all healthcare workers to give answers without the possibility of assuming an evaluation by the researchers. Moreover, due to the self-reporting nature of the SANN-G questionnaire, responses are based on healthcare workers’ subjective interpretation, which may not fully capture the complexity of attitudes or behaviors in real-world practice. Finally, participants were asked to report whether they had ever taken a course on malnutrition, without specifying the nature, duration, or timing of the course. This could be a limitation, as courses of varying lengths and organized by different institutions may have different impacts on attitudes toward older adults’ nutrition.

Future studies should consider longitudinal designs to better understand how education on malnutrition influences attitudes over time and across different healthcare contexts. Expanding the study to include diverse geographical areas and employing mixed-method approaches could provide a more comprehensive understanding of the factors shaping attitudes toward older adults’ nutrition.

## Figures and Tables

**Table 1:** Demographic and education distribution and healthcare workers’ attitudes toward nutrition in older adults and global score in SANN-G.

	Overall (*n* = 1789)
Sex	*n* (%)
Male	219 (12.24)
Female	1227 (68.59)
Missing	343 (19.2)
Age	
20–30 years	162 (9.06)
31–40 years	355 (19.84)
41–50 years	596 (33.31)
51–60 years	336 (18.78)
>60 years	55 (3.07)
Missing	285 (15.9)
Role	
Physician	395 (22.08)
Nurse	254 (14.20)
Nursing assistant	641 (35.83)
Other	149 (8.33)
Missing	350 (19.6)
Did you do an education course on malnutrition?	
Yes	497 (27.78)
No	1084 (60.59)
Missing	208 (11.6)
Positive attitudes per each SANN-G dimension?	
Norms	239 (13.36)
Habits	902 (50.42)
Assessment	716 (40.02)
Intervention	871 (48.69)
Individualization	983 (54.95)
SANN-G global score	
Positive attitudes	420 (23.48)
Neutral attitudes	1012 (56.57)
Negative attitudes	357 (19.96)

**Table 2:** Attitudes toward nutritional care (SANN-G global score) according to sociodemographic characteristics of healthcare workers.

	Negative Attitudes	Neutral Attitudes	Positive Attitudes	χ^2^	*p*-Value
	%	%	%		
Sex				4.89	0.087
Male	24.66	52.05	23.29		
Female	18.34	55.26	26.40		
Age				24.74	0.002
20–30 years	17.90	59.26	22.84		
31–40 years	14.37	56.62	29.01		
41–50 years	20.80	56.38	22.82		
51–60 years	21.73	47.92	30.36		
>60 years	32.73	52.73	14.54		
Role				67.60	<0.001
Physician	25.32	50.63	24.05		
Nurse	9.45	47.24	43.31		
Nursing assistant	23.09	56.79	20.12		
Other	18.79	46.31	34.90		
Did you do a training course on malnutrition?				22.08	<0.001
Yes	17.51	49.09	33.40		
No	19.28	58.39	22.33		

**Table 3:** Factors associated with positive attitudes versus negative or neutral attitudes toward nutritional care (SANN-G global score).

	OR	95% CI	*p*-Value
Sex			
Female vs. Male	1.33	0.92–1.95	0.141
Age (years)			
31–40 vs. 20–30	1.24	0.77–2.02	0.390
41–50 vs. 20–30	0.95	0.60–1.52	0.815
51–60 vs. 20–30	1.46	0.89–2.42	0.137
>60 vs. 20–30	0.70	0.27–1.63	0.427
Role			
Nurse vs. Physician	2.42	1.66–3.55	<0.001
Nursing assistant vs. Physician	0.81	0.59–1.13	0.219
Other vs. Physician	2.14	1.37–3.31	<0.001
Training course on malnutrition			
Yes vs. No	1.54	1.16–2.03	0.002

Notes: OR = Odds Ratio; CI = Confident interval.

## Data Availability

The data presented in this study are available on request from the corresponding author due to privacy and legal reasons.

## Supplementary Materials

The following supporting information can be downloaded at: https://www.mdpi.com/article/10.3390/geriatrics10010013/s1. Table S1: Attitudes toward organizing and implementing mealtimes (norms dimension) according to sociodemographic characteristics of the respondents; Table S2: Attitudes toward patients’ nutritional needs consideration and the right quantities they have to take (habits dimension) according to sociodemographic characteristics of the respondents; Table S3: Attitudes toward the assessment of nutritional status (assessment dimension) according to sociodemographic characteristics of the respondents; Table S4: Attitudes toward interventions needed to manage the disorders linked to malnutrition (Intervention dimension) according to sociodemographic characteristics of the respondents; Table S5: Attitudes toward issues entailed by individualizing meals (Individualization dimension) according to sociodemographic characteristics of the respondents; Table S6: Mean score of SANN-G scale compared by sex, age, role and malnutrition course attendance.

## References

[1] Wolters M., Volkert D., Streicher M., Kiesswetter E., Torbahn G., O’Connor E.M., O’Keeffe M., Kelly M., O’Herlihy E., O’Toole P.W. (2019). Prevalence of malnutrition using harmonized definitions in older adults from different settings—A MaNuEL study. Clin. Nutr..

[2] Nakamura T., Matsumoto M., Haraguchi Y., Ishida T., Momomura S.I. (2020). Prognostic impact of malnutrition assessed using geriatric nutritional risk index in patients aged ≥80 years with heart failure. Eur. J. Cardiovasc. Nurs..

[3] Reinders I., Volkert D., de Groot L., Beck A.M., Feldblum I., Jobse I., Neelemaat F., de van der Schueren M.A.E., Shahar D.R., Smeets E. (2019). Effectiveness of nutritional interventions in older adults at risk of malnutrition across different health care settings: Pooled analyses of individual participant data from nine randomized controlled trials. Clin. Nutr..

[4] Milne A.C., Potter J., Vivanti A., Avenell A. (2009). Protein and energy supplementation in elderly people at risk from malnutrition. Cochrane Database Syst. Rev..

[5] Li M., Zhao S., Wu S., Yang X., Feng H. (2021). Effectiveness of Oral Nutritional Supplements on Older People with Anorexia: A Systematic Review and Meta-Analysis of Randomized Controlled Trials. Nutrients.

[6] Schuetz P., Fehr R., Baechli V., Geiser M., Deiss M., Gomes F., Kutz A., Tribolet P., Bregenzer T., Braun N. (2019). Individualised nutritional support in medical inpatients at nutritional risk: A randomised clinical trial. Lancet.

[7] Strasser B., Grote V., Bily W., Nics H., Riedl P., Jira I., Fischer M.J. (2023). Short-Term Effects of Dietary Protein Supplementation on Physical Recovery in Older Patients at Risk of Malnutrition during Inpatient Rehabilitation: A Pilot, Randomized, Controlled Trial. Healthcare.

[8] Brunner S., Mayer H., Qin H., Breidert M., Dietrich M., Müller Staub M. (2022). Interventions to optimise nutrition in older people in hospitals and long-term care: Umbrella review. Scand. J. Caring Sci..

[9] Khor P.Y., Vearing R.M., Charlton K.E. (2022). The effectiveness of nutrition interventions in improving frailty and its associated constructs related to malnutrition and functional decline among community-dwelling older adults: A systematic review. J. Hum. Nutr. Diet..

[10] James M.K. (2023). The Lack of Nutritional Competency among the Medical Practitioners and Medical Students: A Systematic Review. Eur. J. Nutr. Food Saf..

[11] Crowley J., Ball L., Hiddink G.J. (2019). Nutrition in medical education: A systematic review. Lancet Planet. Health.

[12] Johnston E., Mathews T., Aspry K., Aggarwal M., Gianos E. (2019). Strategies to Fill the Gaps in Nutrition Education for Health Professionals through Continuing Medical Education. Curr. Atheroscler. Rep..

[13] Wojzischke J., van Wijngaarden J., van den Berg C., Cetinyurek-Yavuz A., Diekmann R., Luiking Y., Bauer J. (2020). Nutritional status and functionality in geriatric rehabilitation patients: A systematic review and meta-analysis. Eur. Geriatr. Med..

[14] Castaldo A., Bassola B., Zanetti E.S., Nobili A., Zani M., Magri M., Verardi A.A., Ianes A., Lusignani M., Bonetti L. (2024). Nursing Home Organization Mealtimes and Staff Attitude Toward Nutritional Care: A Multicenter Observational Study. J. Am. Med. Dir. Assoc..

[15] Patel P., Kassam S. (2022). Evaluating nutrition education interventions for medical students: A rapid review. J. Hum. Nutr. Diet..

[16] Cereda E. (2012). Mini nutritional assessment. Curr. Opin. Clin. Nutr. Metab. Care.

[17] Roberts S., Collins P., Rattray M. (2021). Identifying and Managing Malnutrition, Frailty and Sarcopenia in the Community: A Narrative Review. Nutrients.

[18] Bonetti L., Bagnasco A., Aleo G., Sasso L. (2013). Validation of the Staff Attitudes to Nutritional Nursing Care Geriatric scale in Italian. Int. Nurs. Rev..

[19] von Elm E., Altman D.G., Egger M., Pocock S.J., Gøtzsche P.C., Vandenbroucke J.P. (2008). The Strengthening the Reporting of Observational Studies in Epidemiology (STROBE) statement: Guidelines for reporting observational studies. J. Clin. Epidemiol..

[20] Charan J., Biswas T. (2013). How to calculate sample size for different study designs in medical research?. Indian J. Psychol. Med..

[21] Bauer S., Halfens R.J.G., Lohrmann C. (2015). Knowledge and attitudes of nursing staff towards malnutrition care in nursing homes: A multicentre cross-sectional study. J. Nutr. Health Aging.

[22] R Core Team (2024). R: A Language and Environment for Statistical Computing.

[23] Zanini M., Catania G., Ripamonti S., Watson R., Romano A., Aleo G., Timmins F., Sasso L., Bagnasco A. (2021). The WeanCare nutritional intervention in institutionalized dysphagic older people and its impact on nursing workload and costs: A quasi-experimental study. J. Nurs. Manag..

[24] Zanini M., Bagnasco A., Catania G., Aleo G., Sartini M., Cristina M.L., Ripamonti S., Monacelli F., Odetti P., Sasso L. (2017). A Dedicated Nutritional Care Program (NUTRICARE) to reduce malnutrition in institutionalised dysphagic older people: A quasi-experimental study. J. Clin. Nurs..

[25] Thomson K., Rice S., Arisa O., Johnson E., Tanner L., Marshall C., Sotire T., Richmond C., O’Keefe H., Mohammed W. (2022). Oral nutritional interventions in frail older people who are malnourished or at risk of malnutrition: A systematic review. Health Technol. Assess..

[26] Beck A.M., Christensen A.G., Hansen B.S., Damsbo-Svendsen S., Møller T.K. (2016). Multidisciplinary nutritional support for undernutrition in nursing home and home-care: A cluster randomized controlled trial. Nutrition.

[27] Brown L.E., Copeman J. (2008). Nutritional care in care homes: Experiences and attitudes of care home staff. J. Hum. Nutr. Diet..

[28] Mortsiefer A., Löscher S., Pashutina Y., Santos S., Altiner A., Drewelow E., Ritzke M., Wollny A., Thürmann P., Bencheva V. (2023). Family Conferences to Facilitate Deprescribing in Older Outpatients With Frailty and With Polypharmacy: The COFRAIL Cluster Randomized Trial. JAMA Netw. Open.

