# Supplementary material for: Healthcare Workers’ Attitudes Toward Older Adults’ Nutrition: A Descriptive Cross-Sectional Study in Italian Nursing Homes
Source: Geriatrics (Basel). 2025 Jan 16;10(1):13. doi: 10.3390/geriatrics10010013 (PMC11755615; doi:10.3390/geriatrics10010013)
Supplement: Supplementary file 1 [file geriatrics-10-00013-s001.zip › geriatrics-3298511-supplementary.pdf]

Article

Healthcare Workers' Attitudes Toward Older Adults' Nutrition: A Descriptive Cross-Sectional Study in Italian Nursing Homes

Supplementary contingency tables

	Negative attitudes	Positive attitudes	χ2	p-value
	%	%		
**Sex**			0.94	.33
Male	83.56	16.44		
Female	86.06	13.94		
**Age**			15.61	.003
20-30 years	90.74	9.26		
31-40 years	84.22	15.78		
41-50 years	87.25	12.75		
51-60 years	79.76	20.24		
>60 years	90.91	9.09		
**Role**			32.94	<.001
Physician	85.82	14.18		
Nurse	77.16	22.84		
Nurse assistant	89.86	10.14		
Other	76.51	23.49		
**Did you do a training course on malnutrition**			8.48	.003
Yes	81.29	18.71		
No	86.90	13.10		

Table S1: Attitudes towards organizing and implementing mealtimes (norms dimension) according to sociodemographic characteristics of the respondents

	Negative attitudes	Positive attitudes	χ2	p-value
	%	%		
**Sex**			21.62	<.001
Male	63.01	36.99		
Female	45.97	54.03		
**Age**			18.17	.001
20-30 years	51.23	48.77		
31-40 years	44.22	55.78		
41-50 years	49.16	50.84		
51-60 years	47.62	52.38		
>60 years	74.54	25.46		
**Role**			17.61	<.001
Physician	52.15	47.85		
Nurse	39.37	60.63		
Nurse assistant	50.55	49.54		
Other	38.25	61.75		
**Did you do a training course on malnutrition**			1.08	.298
Yes	46.08	53.92		
No	48.89	51.11		

Table S2: Attitudes towards patients' nutritional needs consideration and the right quantities they have to take (habits dimension) according to sociodemographic characteristics of the respondents

	Negative attitudes	Positive attitudes	χ2	p-value
	%	%		
**Sex**			0.15	.695
Male	55.71	44.29		
Female	57.13	42.87		
**Age**			2.67	.614
20-30 years	59.88	40.12		
31-40 years	55.77	44.23		
41-50 years	58.56	41.44		
51-60 years	56.25	43.75		
>60 years	65.45	34.55		
**Role**			29.21	<.001
Physician	59.49	40.51		
Nurse	42.52	57.48		
Nurse assistant	61.93	38.07		
Other	55.70	44.30		
**Did you do a training course on malnutrition**			9.30	.002
Yes	51.51	48.49		
No	59.69	40.31		

Table S3: Attitudes towards the assessment of nutritional status (assessment dimension) according to sociodemographic characteristics of the respondents

	Negative attitudes	Positive attitudes	χ2	p-value
	%	%		
**Sex**			4.72	.030
Male	56.62	43.38		
Female	48.65	51.35		
**Age**			16.28	.003
20-30 years	46.30	53.70		
31-40 years	42.25	57.75		
41-50 years	55.03	44.97		
51-60 years	52.38	47.62		
>60 years	52.73	47.27		
**Role**			33.99	<.001
Physician	55.95	44.05		
Nurse	33.86	66.14		
Nurse assistant	52.26	47.74		
Other	46.31	53.69		
**Did you do a training course on malnutrition**			11.62	<.001
Yes	43.26	56.74		
No	52.49	47.51		

Table S4: Attitudes towards interventions needed to manage the disorders linked to malnutrition (Intervention dimension) according to sociodemographic characteristics of the respondents

	Negative attitudes	Positive attitudes	χ2	p-value
	%	%		
**Sex**			0.36	.548
Male	41.10	58.90		
Female	43.28	56.72		
**Age**			10.59	.032
20-30 years	39.51	60.49		
31-40 years	41.13	58.87		
41-50 years	46.98	53.02		
51-60 years	38.09	61.91		
>60 years	52.73	47.27		
**Role**			9.85	.020
Physician	45.32	54.68		
Nurse	36.61	63.39		
Nurse assistant	47.27	52.73		
Other	39.60	60.40		
**Did you do a training course on malnutrition**			4.14	.042
Yes	38.83	61.17		
No	44.28	55.72		

Table S5: Attitudes towards issues entailed by individualizing meals (Individualization dimension) according to sociodemographic characteristics of the respondents

	Total score	t/F	p-value
**Sex**		-1.279	.20
Male	62.23		
Female	63.31		
**Age**		5.621†	<.001
20-30 years	63.30		
31-40 years	65.13*		
41-50 years	62.07*		
51-60 years	63.03		
>60 years	58.85*		
**Role**		17.872	<.001
Physician	61.61‡		
Nurse	67.26‡		
Nurse assistant	61.57‡		
Other	65.16‡		
**Did you do a training course on mal-nutrition**		3.591†	<.001
Yes	64.79		
No	62.52		

Notes: † = Welch's ANOVA/t-test not assuming equal variances (based on Levene’s test); * = significant difference at post-hoc test between groups 31-40 vs 41-50 and 31-40 vs >60; ‡ = significant difference at post-hoc test between groups nurse vs physician, physician vs other, nurse vs nurse assistant and nurse assistant vs other

Table S6: Mean score of SANN-G scale compared by sex, age, role and malnutrition course attendance
